# Superoxide dismutase activity of serum IgG in acute illness and remission period of schizophrenia

**DOI:** 10.1192/j.eurpsy.2022.733

**Published:** 2022-09-01

**Authors:** I. Mednova, L. Smirnova, A. Vasilieva, N. Krotenko, E. Dmitrieva, A. Semke, S. Ivanova

**Affiliations:** 1 Tomsk National Research Medical Center of the Russian Academy of Sciences, Mental Health Research Institute, Tomsk, Russian Federation; 2 Siberian State Medical University, Department Of Biomedicine, Tomsk, Russian Federation; 3 Mental Health Research Institute, Tomsk National Research Medical Center of the Russian Academy of Sciences, Department Of Endogenous Disorders, Tomsk, Russian Federation

**Keywords:** oxidative stress, schizophrenia, IgG

## Abstract

**Introduction:**

According to recent research violations of the oxidative-antioxidant balance may play an important role in the pathogenesis of schizophrenia, by changing generate, conduct and reproduce a nerve impulse. Antibodies with oxidoreductase activity may be involved in protection against oxidative stress in schizophrenia.

**Objectives:**

The study was to compare the superoxide dismutase (SOD) activity of IgG in patients with acute schizophrenia and during remission.

**Methods:**

The study included 20 patients with acute schizophrenia (mean PANSS total score 94,3±14), 18 people with schizophrenia during remission (mean PANSS total score 54,7±9), and 10 healthy individuals. All participants signed informed consent for the research. Isolation of IgG from blood serum was performed using affinity chromatography on Protein-G-Sepharose columns. The homogeneity of the substances is confirmed by the SDS PAGE method. SOD activity of IgG was carried out spectrophotometrically. Statistical processing was conducted with Statistica v.10.

**Results:**

IgG of schizophrenia patients and healthy individuals have a SOD activity and studied activity is proved to be antibodies intrinsic property. The activity of antibodies in acute schizophrenia was 1.7 times higher than in healthy individuals (p<0.05). In patients with schizophrenia during remission SOD activity of IgG was 2.4 times higher than in healthy individuals (p<0.05).

**Conclusions:**

We can assume that in the conditions of oppression antioxidant activity in schizophrenia patients, antibodies partially take over the function of protecting the body from patients with generalized oxidative stress. *This work is supported by the Russian Scientific Foundation, grant # 18-15-00053P.*

**Disclosure:**

No significant relationships.

